# A purine-rich element in foamy virus *pol* regulates *env* splicing and *gag*/*pol* expression

**DOI:** 10.1186/s12977-017-0337-6

**Published:** 2017-02-06

**Authors:** Rebecca Moschall, Sarah Denk, Steffen Erkelenz, Christian Schenk, Heiner Schaal, Jochen Bodem

**Affiliations:** 1 Institut für Virologie Und Immunbiologie, Julius-Maximilians-Universität Würzburg, Versbacher Str. 7, 97078 Würzburg, Germany; 2 Institute of Virology, Heinrich-Heine-University Düsseldorf, Düsseldorf, Germany

**Keywords:** Splice regulation, Foamy viruses, Branch point, Purine-rich element, RNA export

## Abstract

**Background:**

The foamy viral genome encodes four central purine-rich elements localized in the integrase-coding region of *pol*. Previously, we have shown that the first two of these RNA elements (A and B) are required for protease dimerization and activation. The D element functions as internal polypurine tract during reverse transcription. Peters et al., described the third element (C) as essential for *gag* expression suggesting that it might serve as an RNA export element for the unspliced genomic transcript.

**Results:**

Here, we analysed *env* splicing and demonstrate that the described C element composed of three GAA repeats known to bind SR proteins regulates *env* splicing, thus balancing the amount of *gag*/*pol* mRNAs. Deletion of the C element effectively promotes a splice site switch from a newly identified *env* splice acceptor to the intrinsically strong downstream localised *env* 3′ splice acceptor permitting complete splicing of almost all LTR derived transcripts. We provide evidence that repression of this *env* splice acceptor is a prerequisite for *gag* expression. This repression is achieved by the C element, resulting in impaired branch point recognition and SF1/mBBP binding. Separating the branch point from the overlapping purine-rich C element, by insertion of only 20 nucleotides, liberated repression and fully restored splicing to the intrinsically strong *env* 3′ splice site. This indicated that the *cis*-acting element might repress splicing by blocking the recognition of essential splice site signals.

**Conclusions:**

The foamy viral purine-rich C element regulates splicing by suppressing the branch point recognition of the strongest *env* splice acceptor. It is essential for the formation of unspliced *gag* and singly spliced *pol* transcripts.

**Electronic supplementary material:**

The online version of this article (doi:10.1186/s12977-017-0337-6) contains supplementary material, which is available to authorized users.

## Background

At least three co-transcriptional modifications, namely capping, splicing and polyadenylation are essential to licence mRNA for nuclear export. Since retroviral pre-mRNAs are polycistronic they make extensive use of alternative splicing to regulate viral gene expression. Simple retroviruses, which encode exclusively *gag, pol*, and *env,* express *env* via a spliced transcript; while *gag* and *pol* are translated from the non-spliced genomic RNA. Complex retroviruses, beside the canonical genes, also encode additional regulatory genes whose expression extensively depends on alternative splicing. Thus, splicing needs to be carefully balanced to ensure regulated expression of all viral genes.

Intron borders are defined by conserved sequence elements, termed the 5′ and 3′ splice site (ss), which are recognized by the spliceosome during intron removal [1]. The 3′ss is composed of an invariant AG-dinucleotide, which is preceded by a branch point sequence (BP) and a polypyrimidine tract, both contributing to the intrinsic strength of the 3′ss and hence, efficiency of 3′ss recognition [1]. Usually, the BP is located 10–100 bp upstream of the 3′ss and increases in BP strength promote splice acceptor usage [1]. Furthermore, regulation of alternative splicing can be achieved by the influence of splicing regulatory elements (SREs). These elements represent *cis*-acting binding sites for regulatory proteins, such as serine/arginine-rich proteins (SR-proteins) and heterogeneous nuclear ribonucleoproteins (hnRNPs) acting in a position-dependent manner [2]. It was shown that SR-protein binding enhances splicing only if the SRE was located upstream of the 5′ss, but repressed splicing from a downstream position.

Retroviruses need to promote nuclear export of the unspliced genomic and partially spliced, but still intron-containing mRNAs. However, such mRNAs are usually retained in the nucleus and subsequently degraded. To solve this dilemma, complex retroviruses such as the Human Immunodeficiency Virus type 1 (HIV-1), the Mouse Mammary Tumour Virus, and Human T cell leukemia Virus, encode export-mediating proteins, which bind intron-containing mRNAs and interact with the cellular karyopherin CRM1/exportin 1 [3–7]. These ternary complexes are then transported through the nuclear pores and viral RNAs are released into the cytoplasm. Simple retroviruses, such as Mason Pfizer Monkey virus use a constitutive transport element, which directly interacts with the nuclear export factor 1 [8–10].

Foamy viruses belong to complex retroviruses since they encode regulatory proteins in the 3′ region but still constitute a separate subfamily [11, 12]. The foamy viral genome encodes four central purine-rich elements, abbreviated as A, B, C and D, localized in the integrase encoding region of *pol* [13]. The D element was shown to function as an internal polypurine tract during reverse transcription, whereas the A and B elements are required for protease dimerization and activation [13, 14]. Foamy viral gene expression is distinct from other retroviruses (for review see [15, 16]) since they express *pol* from a spliced transcript [17, 18]. Furthermore, the major splice donor is located in the R-region of both LTRs, which is unique among retroviruses [19, 20]. Although splice sites have been mapped in the prototype foamy virus (PFV) (previously called human foamy virus [12]) and in the feline foamy virus, so far the regulation of foamy viral splicing has not been systematically investigated [20, 21].

Furthermore, foamy viruses use a variation of the cellular mRNA export pathway, which has been also described for CD83 mRNA and heat-shock protein encoding transcripts [22, 23]. The cofactors karyopherin CRM1 and ANP32A/B support the nuclear export of the foamy viral genomic and *pol* transcripts [15, 24]. However, the RNA element interacting with the cellular export machinery has not yet been determined. Recently, a mutation of the internal purine-rich C element within *pol* has been described which resulted in suppression of *gag* expression and lack of infectivity [13]. Thus, this element could serve as part of an export-mediating RNA structure or might influence splicing. Here, we functionally characterize the mechanism which leads to suppression of *gag* and *pol* expression.

## Results

### The internal polypurine-rich element is not responsible for RNA export

**Fig. 1:**
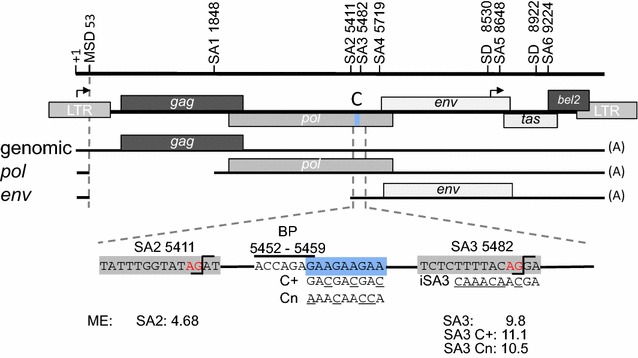
Scheme of the LTR-derived transcripts of PFV. The position of the C element is highlighted in *blue*. Exchanged nucleotides of the C element and SA3 sequences are *underlined*. The MaxEnt scores (ME) of SA2, SA3 and of the SA3 mutations C+ or Cn are depicted below the scheme. *Arrows* indicate positions of the LTR and the internal promoter. *SA* splice acceptor, *SD* splice donor, *MSD* major splice donor

**Fig. 2:**
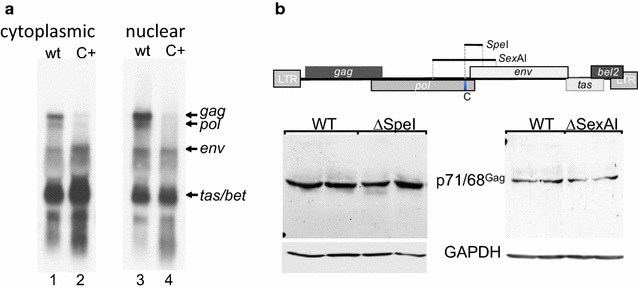
Modification of the C element abolishes *gag* and *pol* expression but C element is not essential for RNA export. **a** Subcellular distribution of foamy viral transcripts was analysed by northern blotting using a *tas*-specific probe. *bet* is expressed from a spliced transcript encompassing parts of the *tas and bel2* gene [15, 20]. **b** Influences of the *pol* elements located in the *Spe*I- and S*exA*I-fragments on Gag expression were analysed by Western blotting with monoclonal anti-Gag antibodies. The position of the C element is indicated (*blue box*). Detection of GAPDH served as loading control. Samples were analysed in independent duplicates

Previously an RNA element located in PFV *pol* was shown to be able to functionally replace the Rev/Rev-responsive element for HIV-1 Gag expression [25]. Since the C+ mutation within the purine-rich C element of the foamy viral *pol* region (Fig. 1) was reported to cause loss of *gag* expression [13], we addressed the question of whether the foamy viral C element might be an essential part of the nuclear export element. Therefore, we introduced the C+ mutations in the PFV proviral plasmid pHSRV13 (Fig. 1), transfected BHK21-ll cells and 48 h later analysed the subcellular viral RNA distribution. To this end, nucleic and cytoplasmic RNA preparations were analysed by northern blotting and viral RNAs were detected with a *tas* probe, encompassing the complete *tas* encoding sequence, as described before (Fig. 2a) [19]. This Northern blotting analysis was repeated twice with independent samples. Strikingly, the C+ mutation of the purine-rich element clearly led to lower amounts of viral *gag* and *pol* transcripts in both subcellular fractions (Fig. 2a, cf. lanes 1 with 2 and 3 with 4). Overall, the cytoplasmic amounts of *tas/bet* and *env* transcripts were increased by the three A to C substitutions within the C element (pHSRV13C+). The impact of the C+ mutation on *tas/bet* expression was not further addressed in this report. However, our results underline the strong reduction in Gag protein previously observed by Peters et al. [13], but excluded the nuclear RNA export pathway as the cause of Gag suppression.

To further determine whether the region encoding the C element is essential for *gag* expression, we analysed two deletion mutants where the *Spe*I or the *SexA*I fragments of pHSRV13 harbouring the C element and known *env* 3′ss had been removed (Figs. 1, 2b). Interestingly, both deletion mutants expressed *gag* in similar amounts compared to cells transfected with the wild-type pHRSV13 plasmid; confirming that the purine-rich C element is not involved in the nuclear export of unspliced genomic RNA.

These results suggest that oversplicing at *env* 3′ss caused by mutating the C element might have shifted the splicing pattern towards viral transcripts where the first intron had been removed. Additionally, these results demonstrate that neither these splice acceptors nor the C element itself are a prerequisite for Gag expression. Thus, we determined the splicing pattern of *gag*, *pol* and *env* transcripts to analyse whether the mutation of the purine-rich C element would change the efficiency of intron removal necessary for *env* mRNA processing.

### Identification of a new major *env* 3′ splice site

**Fig. 3:**
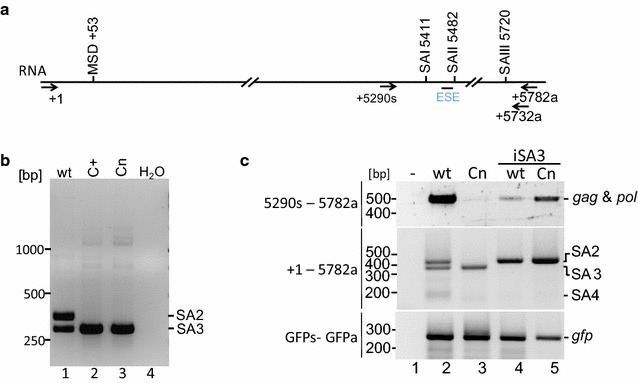
PFV *env* is expressed by two major transcripts spliced to 3′ss–modification of the C element results in exclusive splicing to SA3. **a** Scheme of the locations of splice sites, of the C element and position of RT-PCR primers. **b** Detection of *env* 3′ splice sites by RT-PCR using a primer +1s and 5732a located downstream of the *env* start codon. Amplicons were separated on agarose gels and splice sites determined by nucleotide sequencing. **c** The mutation of the C element decreases genomic and *pol* transcripts. Foamy virus *env* is expressed by three transcripts alternatively spliced to SA2, SA3, and weakly to SA4. The Cn mutations results in splicing to SA3 and a reduction of genomic and pol mRNAs. The inactivation of SA3 (iSA3) partially recovers *gag* and *pol* expression

In order to analyse the influence of the C element on *env* splicing, BHK21-ll cells were transiently transfected with the proviral plasmids pHSRV13 or pHSRV13C+. RNAs were isolated and *env* transcripts were identified by RT-PCR using a primer located in exon 1 and a primer located 302 nucleotides downstream of the *env* start codon (+5732a) (Fig. 3a). Amplicons covering the 5′end of the *env* transcripts were separated on an agarose gel and two distinct exon–exon-junctions due to alternatively used 3′ss were detected (Fig. 3a, lane 1). Nucleotide sequencing revealed that one of the products corresponded to a spliced *env* mRNA with an already published intrinsically very strong 3′ss (MaxEnt score (ME) of 9.84) at position 5482 (aka 5478 in [20]), here referred to as SA3 (Fig. 1). Surprisingly, a second major *env* transcript generated by using a much weaker (ME 4.68), so far unknown *env* 3′ss at position 5411 was also identified (here referred to as SA2) (Fig. 3a, lane 1). The apparent comparable splice site usage of two competing sites in close proximity to each other suggests that the much stronger SA3 might be repressed.

A previously described 3′ss at 5720 [20] remained barely detectable by RT-PCR (SA4) (ME 4.87) (Fig. 3b, lane 2), so that the splicing pattern of PFV *env* transcripts resembles the splicing pattern of the feline foamy virus [21], i.e. expressing two alternatively spliced *env* transcript isoforms by using one of two competing 3′ss. The 5′ss of a previously described non-coding transcript ([20]: “transcript 2”) using the 3′ss at 5411, however, could not be detected throughout this analysis.

### Mutation of the C element shifted *env* splice site usage entirely towards SA3

To characterise the influence of the purine-rich element C on *env* 3′ss usage, pre-mRNA splicing was also analysed in the mutant provirus. Two days post-transfection, RNA was isolated and subjected to RT-PCR analyses. While SA2 and SA3 had been used with almost equal efficiency in the wild-type context, in the case of the C+ mutant (ME 11.1) selection entirely shifted towards SA3 (Fig. 3b, cf. lanes 1 and 2). Since the C+ mutation only barely impacted the difference in intrinsic splice site strength between SA2 and SA3 [∆ME 5.16 SA2/SA3 vs. 6.42 SA2/SA3 C+ (Fig. 1)], it suggested that the splice site switch might be caused by liberating a repressive activity on SA3 usage.

To exclude effects on splicing by the specific sequence introduced by Peters et al. [13], the purine-rich C element was replaced by a “neutral” sequence (Cn) (ME 10.5), which neither forms secondary RNA structures nor binds splicing regulatory proteins leading to pHSRV13Cn (Figs. 1, 3b) [26, 27]. Moreover, this neutral sequence altered the difference in intrinsic splice site strength between SA2 and SA3 even less than the C+ mutation (∆ME 5.16 SA2/SA3 vs. 5.84 SA2/SA3 Cn).

BHK21-ll cells were transfected with pHSRV13Cn, however, a similar shift towards splicing at SA3 was observed (Fig. 3c, cf. lanes 1—3). These results indicated that mutation of the purine-rich C element, without largely impacting the intrinsic splice site strength of SA3, was sufficient to dramatically influence SA2/SA3 splice site choice towards the intrinsically stronger site SA3.

To confirm that the C mutations did not inactivate SA2 but simply set aside the repression exerted on SA3; and thus prevent splice site competition, we inactivated SA3 (iSA3) in both proviral plasmids, pHSRV13 and pHSRV13Cn (Figs. 1, 3c). This inactivation resulted in an expected shift towards splicing at SA2 for both proviruses, indicating that SA2 was still functional in the context of the C-mutations. Additionally, a decrease in intron-containing *gag/pol* transcripts was observed (Fig. 3c, lane 4). This increase in the amount of transcripts spliced to SA2 and the decrease in *gag/pol* transcripts could be explained by an oversplicing phenotype, as a result of the missing splice site competition between SA2 and SA3. These results confirmed that suppression of retroviral splice site strength in particular is critical for balancing expression of all viral proteins.

### The poly-purine rich element is essential for *gag* and *pol* expression

**Fig. 4:**
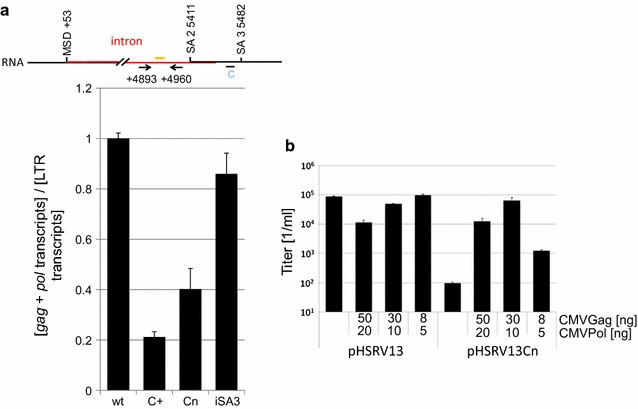
**a** *env* intron-containing *gag* and *pol* transcript amounts are decreased by the C+/Cn modifications. Changes in the splicing patterns of genomic and *pol* transcripts were analysed by RTqPCR and expressed as ratios of intron-containing transcripts divided by all *env* transcripts [*env* intron transcripts]/[*env* spliced + *env* intron]. *Bars* represent the mean of three independent samples and *error bars* the standard deviation. Locations of the primers (*arrows*) and probes (*yellow line*) are depicted above the panel. **b** Cotransfection of pCMVgag and -pol rescues infectivity of pHSRV13Cn. Viral titers of transfected BHK21 cells were determined on BHK21-ll cells by intracellular β-Galactosidase staining

To quantify the relative abundance of alternatively spliced transcripts, RNA from cells either transfected with wild-type or mutant provirus plasmids were subjected to real-time RT-PCR analyses. Taqman-PCR was used for the detection of unspliced genomic and *pol* transcripts, quantifying transcripts containing the *env* intron (positions 4893 and 4960) (Fig. 4a). Results were obtained from three independent experiments and analyses were repeated at least four times. Dilutions of RNAs from cells transfected with pHSRV13 were used as the reference standard curve for the relative quantification of all samples. Both pHSRV13C+ and pHSRV13Cn led to a fivefold and 2.5 fold decrease in relative amounts of gag/*pol* transcripts, respectively (Fig. 4a), supporting the Northern blot results (cf. Fig. 2). On the other hand, the additional inactivation of SA3 in the context of the Cn mutation (pHSRV13Cn iSA3) restored *gag* and *pol* expression almost to wild-type levels and confirmed the experiments of the previous experiment (Fig. 3c). These experiments suggest that weakening SA3 through the purine-rich C element controls retention of the *env* introns, i.e. the unspliced genomic and *pol* mRNA transcripts. In summary, this experiment provides further evidence that the purine rich C element represents a splicing regulatory element (SRE) controlling removal of the *env* intron to ensure processing of required amounts of *gag* and *pol* transcripts.

As these results indicate that genomic and *pol* transcripts were affected by the mutation of the C element, we analysed whether cotransfection of *gag* and *pol* expression plasmids would rescue decreased virion production observed with the mutations of the C element *in trans*. BHK21-ll cells were transfected with either pHSRV13 or pHSRV13Cn and increasing amounts of pCMVgag and pCMVpol (Fig. 4b). Viral titers of the cellular supernatants were determined using BHK21-ll indicator cells, which contain an integrated LacZ gene controlled by the PFV LTR promoter. Infected cells express the viral transactivator Tas, which subsequently activates the LTR promoter. The Cn mutation resulted in a drop in titers of three orders of magnitude (Fig. 4b). Cotransfection of codon-optimized *gag* and *pol* expression plasmids resulted in titers comparable to pHSRV13 demonstrating that the impaired infectivity of pHSRV13Cn could be restored if sufficient Gag and Pol amounts were provided. The RT-qPCR (Fig. 4a) showed a moderate influence of Cn on genomic/pol transcript amounts (factor 0.4). However, these differences resulted in a huge decrease in Gag protein expression [13] indicating that Gag protein amounts were more affected than the expression of the genomic transcript. Yet, the cotransfection experiment indicated that compensation of decreased *gag/pol* expression was sufficient to rescue infectivity. This supports the hypothesis that the genomic transcript is efficiently packaged by the Gag protein derived from the transfected codon-optimized expression vector. The observed slightly reduced viral titers in the supernatants of cells cotransfected with *gag*, *pol* and pHSRV13Cn compared to pHSRV13 might be a consequence of the lower expression of the genomic transcript (Fig. 4a).

### The amounts of the *env* transcripts are controlled by the C element

**Fig. 5:**
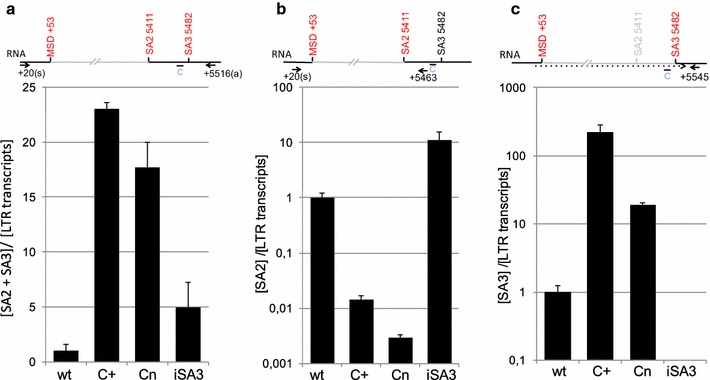
**a** Mutation of the C element leads to higher *env* amounts. Expression was determined by qRTPCR, calculated as ratio of spliced transcripts divided by all transcripts in *env* [env spliced]/[LTR transcripts] and normalized on pHSRV13. *Bars* represent the mean of three independent samples and *error bars* the standard deviation. Locations of the primers are depicted above the panels. **b** The C element increases SA2 strength. The usage of primer 5463 excludes amplification of SAIII splice transcripts, while other transcripts, such as genomic and *pol,* are excluded by the extension time. *Bars* represent the mean of three independent sample RNAs and *error bars* represent the standard deviation. **c** The modification of the C element leads to almost exclusive SA3 usage. The primer 42 spans the MSD-SAIII splice site junction and ensures selective amplification of SAIII spliced transcripts. *Bars* represent the mean of three independent samples and *error bars* the standard deviation

Since the purine-rich C element represses SA3 usage (cf. Fig. 3), leading to *env* intron retention, the inactivation of this SRE should consequently impact SA3 usage resulting in increased amounts of *env* transcripts. Thus, we quantified the amounts of both *env* transcripts spliced at SA2 and SA3 by RT-qPCR using primer pair +20(s) and +5516(a) (Fig. 5a). The Taqman-probe used for detection of the *env*-spliced transcripts was located upstream of the major 5′ss (MSD +53), whereas the antisense primer was placed downstream of SA3 at nucleotide 5516 (Fig. 5a). Dilutions of RNA of cells transfected with pHSRV13C+ were used as reference standard curve for the relative quantification of all samples. As expected, *env* transcripts were drastically increased following mutation of the C element; for C+ by more than 20 fold, and for Cn by ~18 fold compared to wild-type (Fig. 5a). These results confirmed that the SRE C element balances the ratio between *gag* and *pol* on one hand and spliced *env* transcripts on the other. Of note, inactivation of SA3 (iSA3), thus liberating splice site competition, only moderately (fivefold) increased SA2 usage confirming the relative weakness of SA2 compared to SA3.

To differentiate between SA2 and SA3 usage, we refined the RT-qPCR analysis using primers specific for SA2 or SA3 spliced transcripts. To determine the amounts of RNAs spliced to SA2 the antisense primer was placed upstream of SA3 at position 5463 (Fig. 5b). These analyses showed that the C+ and Cn mutation led to a 70 fold and 340 fold decrease in relative SA2 *env* transcript levels, respectively (Fig. 5b). Based on these results we hypothesised that the intrinsically much stronger SA3 splice site is repressed by the purine-rich C element. In line with this hypothesis, the C mutations strengthened SA3 so that it outcompeted SA2 usage (Figs. 3a, b, 5c). The inactivation of SA3 (iSA3) in the context of Cn increased the amounts of *env* transcripts spliced at SA2, probably due to elimination of splice site competition indicating that Cn promotes splicing to SA3. Finally, we quantified SA3 specific transcripts by using a sense primer spanning the MSD/SA3 exon–exon-junction. The C+ mutations enhanced splicing at SA3 up to more than 225 fold, whereas the Cn mutations resulted in an approx. 20 fold increase. The inactivation of SA3 resulted in amounts of SA3 spliced transcript below the detection limit, demonstrating the specificity of the reaction. Our results confirm that indeed the observed increase in *env* transcripts (Fig. 5a) upon inactivating the SRE C element was mainly due to an increase in SA3 usage (Fig. 5c). These results provide evidence that suppression of splicing to SA3 is required for *gag* and *pol* expression.

### The purine-rich C element impairs branch point recognition

Purine-rich elements have long been described as binding sites for splicing regulatory proteins in cellular and viral genes, e.g. the native repetitive GAA nucleotides within the calcitonin/CGRP pre-mRNA [28] or the HIV-1 exon 5 GAR splicing enhancer [29, 30]. We analysed whether the purine-rich C element might impair recognition of the SA3 branch point, thereby causing the observed apparent decrease in SA3 splice site strength. This would adequately explain the observed comparable splice site usage in the competing wild-type situation and exclusive SA3 usage following mutation of the interfering binding site of the GAA repeats of the C element (cf. Fig. 3b).

**Fig. 6:**
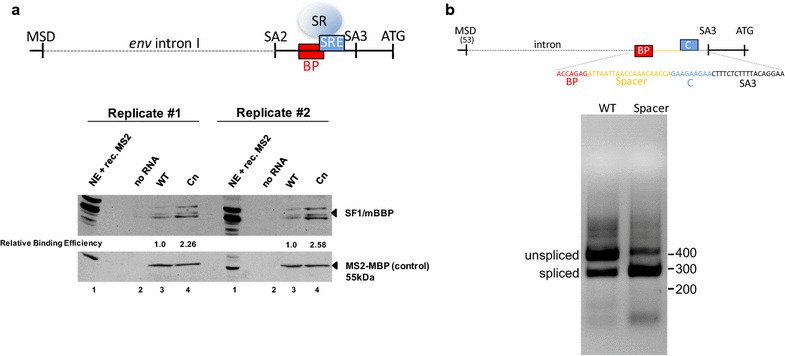
Analyses of wild-type and Cn SA3 recognition by determination of RNA-bound spliceosomal SF1/mBBP. **a** In vitro synthesized and immobilized RNAs encoding a M2 stem loop followed by wild-type or Cn sequences were incubated with HeLa nuclear extracts. Recombinant MS2-MBP (1 µg) was added to monitor immobilized RNA amounts. Bound proteins were visualized by Western blotting analyses using SF1/mBBP or MBP-antibodies and quantified. Lane 1 represents 10% of the load. **b** Separation of BP and SRE sequences by a spacer enhances SA3 splicing. LTR and SA3 were cloned in pGL3. A spacer of 20 nucleotides separated BP and the C element and splicing was analysed by RTPCR. A scheme of the spacer plasmid is depicted above the panel. *SR* serine-arginine-rich, protein, *SRE* splice regulatory sequence

To analyse whether SA3 branch point recognition by binding of SF1/mBBP is negatively regulated by the immediately adjacent or even overlapping purine-rich C element, we performed RNA affinity chromatography assays with RNA substrates containing the branch point sequence with either wild-type C or Cn mutant element sequences. To this end, oligonucleotides were covalently bound to agarose beads and incubated with HeLa nuclear extract (Fig. 6a). Notable, both RNA substrates contained a single RNA recognition site for the bacteriophage MS2 coat protein. This allowed us to control for comparable precipitation efficiencies by adding recombinant MS2 coat–Maltose binding (MBP) fusion protein. Beads were washed and the bound proteins were eluted from the RNAs by incubation at 95 °C for 10 min in protein sample buffer. Precipitated SF1/mBBP and MS2-MBP proteins were determined by Western blotting (Fig. 6a). As it was expected from an increased activation of SA3 due to the disruption of the C element, we could detect higher levels of SF1/mBBP precipitating with Cn mutant RNAs (>2.2 fold). This indicated that the C element is necessary to avert unbalanced high SA3 BP recognition. Since generally a BP is not tightly distance restricted from the 3′ss, we sought to underline our hypothesis by simply separating the SA3 BP and the GAA repeats of the C element by a spacer oligonucleotide (Fig. 6b). Following the insertion of a 20 nt spacer between the BP and the GAA repeats, BHK21-ll cells were cotransfected with the reporter plasmid and a *tas* expression plasmid and transcript levels were analysed by RT-PCR. This analysis showed that separating both sequences led to a switch of the ratio of unspliced and spliced *env* SA3 transcripts (Fig. 6b, cf. WT and Spacer) confirming our hypothesis that the purine-rich C element interferes with SF1/mBBP binding at the branch point sequence, and thus regulates splicing at SA3.

## Discussion

Foamy viruses are complex retroviruses, which regulate gene expression by unique features, such as a spliced *pol* transcript and an internal promoter directing expression of the viral *trans*-activator and the APOBEC-antagonising protein Bet [12, 15, 17, 18, 31, 32]. Previous studies identified up to 16 differentially spliced foamy viral transcripts in infected cells [20, 21]. Here, we re-analysed *env* splicing and could confirm most previously identified *env* splice acceptors. Moreover, PFV *env* is expressed by differential splicing using a newly identified *env* 3′ss (SA2) at nucleotide 5411 and SA3 at nucleotide 5482. However, our RT-PCR analyses showed that the genomic location of PFV *env* SAs are similar to those of feline foamy virus [21]. This conservation is in line with other studies showing that molecular mechanisms are strikingly similar among foamy viruses [12].

Alternative splicing of retroviral pre-mRNAs requires complex regulation to ensure splice site enhancement as well as repression during the viral replication cycle. In the HIV-1 pre-mRNA several splice enhancers and silencers have been identified, which fine-tune exon definition and the ratio between viral transcript isoforms. Alternative splicing of the foamy viral LTR-derived genomic, *pol* and *env* transcripts is balanced between a single 5′ss in the LTR and four 3′ss. All of these 3′ss, *pol* (SA1, ME 5.47), *env* SA2 (ME 4.68), and *env* SA4 (ME 4.87), represent intrinsically weak splice sites compared to SA3 (ME 9.84). Thus, silencing of SA3 is a prerequisite to maintain both intron retention of the genomic RNA and *gag* and *pol* expression. The foamy viral *env* is expressed via two transcripts with the same coding capacity using weak 3′ ss (SA2) and a repressed strong 3′ss (SA3) to allow on one hand expression of *pol* and of the genomic RNA and to ensure at the same time sufficient *env* amounts. The suppression of SA3 is mediated by the C element with a GAA repeat sequence identical to the purine-rich splicing enhancer identified in the human calcitonin/CGRP pre-mRNA [28]. Its location between the two *env* 3´ss is conserved in foamy viruses of chimpanzee and felines underlining its importance (e.g. FFV: SA2 6036; SA3 6307; GAA repeat 6057-6065).

Here, we identified a novel splicing regulatory sequence (SRE) overlapping the BP of the *env*-specific 3′ss SA3. This sequence is necessary for suboptimal recognition of the BP by SF1/mBBP, thereby permitting retention of the *env* intron and formation of unspliced *gag* and singly spliced *pol* transcripts. It has been described that SR protein binding sites promote exon definition, which is in-line with the effects of the C element on SA2 described in this report. SR proteins can lead to exon inclusion by influencing 5′ss selection even when they bind to an intronic position [33–35]. They have been shown to enhance splicing of HPV, SV40 and HIV-1 [36–38]. For example overexpression of SRSF1 leads to complete splicing of the HIV-1 genomic RNAs to *vpr* mRNAs [36]. Nevertheless, we report here a strong suppression of splicing to a downstream splice acceptor (SA3) by the C element. Interestingly, our findings recapitulate a previous study demonstrating that an intronic splicing silencer negatively regulates the access of general splicing components to the BP of the HIV-1 *env*-specific splice acceptor SA7 [39]. Accordingly, both viruses seem to exploit a functionally common mechanism for the maintenance of an appropriate balance between intron-less and intron-containing viral mRNAs. While it was shown for the HIV-1 intronic silencer sequence that it is bound by hnRNP A1 (central motif of the hnRNP A1 binding site (UAGG[G/A]AG) [40, 41]), the “GAA” rich sequence of the foamy viral C element rather excludes hnRNP binding such as A1 or D [42], but suggests an association with a member of the SR protein family which have demonstrated to bind purine-rich sequences (e.g., the HIV-1 GAR element [29]. Recently, it was reported that SR proteins act negatively on MDM2 splicing by inducing exon 11 skipping [43]. The SR protein binding site of MDM2 is located in the skipped exon 11. Furthermore, SR proteins have been documented to act as splicing repressors when bound to an intron [2], also by specifically interfering with the recognition of the BP [44].

## Conclusions

Retroviruses use alternative splicing to regulate their gene expression. We show that a purine-rich element represses the strongest *env* splice acceptor and promotes *env* intron retention. Thus, this regulatory sequence is essential to balance splice acceptor usage and regulates *gag, pol* and *env* amounts.

### Methods

#### Plasmids and mutants

The pHSRV13 proviral plasmid has been described previously [45]. Mutations of the C element and SA3 were generated by site-directed mutagenesis. A detailed description of primers can be found in the Additional file 1. The mutants were introduced into a subcloned *PacI/BspEI* fragment re-inserted into pHSRV13. The *Spe*I or *SexA*I fragments were removed by restriction digestion. Codon optimized *gag* [14] and *pol* [46] expression plasmids were used to restore pHSRV13 infectivity. The pGL3-SA3 splice reporter was constructed by inserting SA3 into the pGL3-LTR plasmid (pGL3-LTR: PFV LTR inserted into the *Kpn*I and *Xho*I sites of pGL3) [19]. Finally, the 20 bp spacer was inserted by site directed mutagenesis.

#### Infectivity assays

Cell culture supernatants were collected, centrifuged (1500 rpm, 5 min) to remove infected cells and titrated in serial dilutions on BHK21-ll cell (96-well plate, 104 cells per well). BHK21-ll cells are BHK21 cells carrying a stably integrated copy of a foamy virus LTR promoter driving a *lacZ* gene [47]. PFV infection leads to the expression of the viral transactivator Tas, which subsequently activates the LTR promoter. Two days post infection BHK21-ll cells were fixed with ice-cold methanol/acetone for 5 min followed by a β-galactosidase stain, using X-gal as substrate. Blue cells were counted and viral titers were subsequently calculated. The assays were performed in at least three independent biological experiments in triplicate and standard deviations were calculated.

#### Northern blotting

For northern blotting, 4 × 10^5^ BHK21-ll cells were cotransfected with 1 µg pHSRV13 (or derivatives) and 0.5 µg peGFPC1. The preparation of total or fractionated RNA was performed as previously described [24]. RNAs (5 µg) were loaded onto a 1% agarose gel and transferred onto a Hybond-N + membrane (Amersham) by capillary transfer. The blots were hybridized overnight at 60 °C to a *tas*-specific probe, encompassing the complete *tas* reading frame (activity >10^7^ cpm), labelled by PCR. The blots were re-hybridized to a human *GAPDH* gene (nucleotides 1011–1310) probe.

#### RT-PCR

RNAs were reverse transcribed with MLV-RT (Promega) and transcript specific primers. cDNAs were amplified with Taq DNA polymerase (NEB) according to the manufacturers’ protocol.

#### RT-qPCR

Probes and primers were designed with the Roche Universal Probe Library Assay Design Center software (https://lifescience.roche.com/webapp/wcs/stores/servlet/CategoryDisplay?catalogId=10001&tab=&identifier=Universal+Probe+Library&langId=-1#tab-3) (Roche, Mannheim). RNA amounts were quantified with the GoTaq^®^ Probe 1-Step RT-qPCR System (Promega) and a Lightcycler96 (Roche, Mannheim) instrument. All assays were performed with at least three independent RNA samples in triplicates and were repeated three times. Input RNA amounts were normalized on *gapdh* expression as determined by RT-qPCR. Primer and probes are listed in the Additional file 1. Dilutions of three independent RNAs of the samples with highest expression of the respective transcript were used for standardisation curves. Relative expression levels were calculated with the Lightcycler96 software according to the manufacture’s description. The relative *env* intron amounts were calculated as ratios of intron-containing transcripts divided by all transcripts in *env* [*env* intron transcripts]/[LTR transcripts] and the *env* splice transcripts as ratios of spliced transcripts divided by all transcripts in *env* [env spliced]/[LTR transcripts]. The transcript amounts were normalized to wild-type amounts.

#### Sequence analysis

The analyses of the strength of splice sites and the localization of splice enhancer or silencer elements were performed with the human splice finder program (http://www.umd.be/HSF3/). The strength of the 3′ss was calculated using the MaxEntScore algorithm (http://genes.mit.edu/burgelab/maxent/Xmaxentscan_scoreseq_acc.html) [48].

#### Western blotting

BHK21-ll cells were transfected and harvested two days post transfection. Western blotting analyses of cellular lysates were performed using Gag-specific monoclonal antibodies (SGG1) [24].

#### RNA pull-down

For RNA affinity chromatography, wild-type and mutant substrate RNAs were synthesized (Wild-type 5′-AAG TAC GAA TTT CCT GTA AAA GAG AAA GTT CTT CTT CTC TGG TCA AGT CAA GTG TAT CTT ACA TGG GTG ATC CTC ATG TCC TAT AGT GAG TCG TAT TA-3′ Mutant: 5′-AAG TAC GAA TTT CCT GTA AAA GAG AAA GTG GTT GTT TTC TGG TCA AGT CAA GTG TAT CTT ACA TGG GTG ATC CTC ATG TCC TAT AGT GAG TCG TAT TA-3′). In vitro synthesized and immobilized RNAs encoding a MS2 stem loop followed by wild-type or Cn sequences and SA3 were incubated with HeLa nuclear extracts (NE) in buffer D (20 mM HEPES–KOH [pH 7.9], 5% glycerol, 0.1 M KCl, 0.2 mM EDTA, 0.5 mM DTT). Recombinant MS2-MBP (1 µg) was added to monitor immobilized RNA amounts. Bound proteins were visualized by western blot analyses using SF1/mBBP (Proteintech, Germany) or MPB-antibodies (Tetracore) and quantified with the ImageJ software version 1.46 (http://imagej.net).

## Additional file

**Additional file 1.** Primers and probes.
