# Supplementary material for: A purine-rich element in foamy virus pol regulates env splicing and gag/pol expression
Source: Retrovirology. 2017 Feb 6;14:10. doi: 10.1186/s12977-017-0337-6 (PMC5294762; doi:10.1186/s12977-017-0337-6)
Supplement: Supplementary file 1 — Additional file 1. Primers and probes. [file 12977_2017_337_MOESM1_ESM.docx]

**Supplementary materials**

**Primer and Probes.**

| **Primer** | **5’ -> 3’** | **Probe No.** |  |
| --- | --- | --- | --- |
| PFV env SAII spez a | CAGGAGACCAGGAACGAGAG | 19 | 5545 |
| PFV env SAII spez s | CTCCAGGTTTGGAAATTCGT | 19 | 42 |
| PFV env SAI a | TTCTTCTCTGGTCAAGTCAAGTGTA | 80 | 5463 |
| PFV env intron a | CAAAAGGTTTTTGAGGCCTATC | 93 | 4960 |
| PFV env intron s | AATCACAAATGCTTCCAACAAA | 93 | 4893 |
| PFV env SAII+SAI a | GGGGTGGATGGATGGTATAA | 80 | +5516 |
| PFV env SAII+SAI s | CTGCGTCGAGAGTGTACGAG | 80 | +20 |
| BHK gapdh a | AAGACGCCAGTAGACTCCACA | 159 |  |
| BHK gapdh s | ATCCCACCAACATCAAATGC | 159 |  |

| Probes | Nucleotide position |
| --- | --- |
| Nr. 93 | 4989 - 4996 |
| Nr. 80 | 112 - 119 |
| Nr. 19 | 5587 - |

**RT-PCR AND RTqPCR:**

| **Primer** | **5’ -> 3’** | **Probe Nr.** |  |
| --- | --- | --- | --- |
| PFV env SAIII spez a | CAGGAGACCAGGAACGAGAG | 19 | 5545 |
| PFV env SAIII spez s | CTCCAGGTTTGGAAATTCGT | 19 | 42 |
| PFV env SAII a | TTCTTCTCTGGTCAAGTCAAGTGTA | 80 | 5463 |
| PFV env intron a | CAAAAGGTTTTTGAGGCCTATC | 93 | 4960 |
| PFV env intron s | AATCACAAATGCTTCCAACAAA | 93 | 4893 |
| PFV env SAII+SAIII a | GGGGTGGATGGATGGTATAA | 80 | +5516 |
| PFV env SAII+SAIII s | CTGCGTCGAGAGTGTACGAG | 80 | +20 |
